# Integrated Care Improved Cognitive ability in Older Chinese Patients with Impaired Intrinsic Capacity

**DOI:** 10.1093/geroni/igaf122.2868

**Published:** 2025-12-31

**Authors:** Ping He, Yi Zhang, Aihong Liu, Bei Cheng, Tangmeng Guo, Jing Ge, Yang Zhao

**Affiliations:** Department of Geriatrics, Union Hospital, Tongji Medical College, Huazhong University of Science and Technology, 1277 Jiefang Avenue, Wuhan 430022, Hubei, China, Wuhan, Hubei, China (People’s Republic); Department of Geriatrics, Union Hospital, Tongji Medical College, Huazhong University of Science and Technology, 1277 Jiefang Avenue, Wuhan 430022, Hubei, China, Wuhan, Hubei, China (People’s Republic); Department of Geriatrics, Union Hospital, Tongji Medical College, Huazhong University of Science and Technology, 1277 Jiefang Avenue, Wuhan 430022, Hubei, China, Wuhan, Hubei, China (People’s Republic); Department of Geriatrics, Union Hospital, Tongji Medical College, Huazhong University of Science and Technology, 1277 Jiefang Avenue, Wuhan 430022, Hubei, China, Wuhan, Hubei, China (People’s Republic); Department of Geriatrics, Union Hospital, Tongji Medical College, Huazhong University of Science and Technology, 1277 Jiefang Avenue, Wuhan 430022, Hubei, China, Wuhan, Hubei, China (People’s Republic); Department of Geriatrics, Union Hospital, Tongji Medical College, Huazhong University of Science and Technology, 1277 Jiefang Avenue, Wuhan 430022, Hubei, China, Wuhan, Hubei, China (People’s Republic); Department of Geriatrics, Union Hospital, Tongji Medical College, Huazhong University of Science and Technology, 1277 Jiefang Avenue, Wuhan 430022, Hubei, China, Wuhan, Hubei, China (People’s Republic)

## Abstract

**Objectives:**

This study aims to investigate the effectiveness of a integrated care model for elderly patients led by geriatric care experts, in order to provide information for the management of intrinsic capacity in older Chinese patients.

**Methods:**

Between June and December 2021, 60 elderly patients with impaired intrinsic capacity were recruited from the Department of Geriatrics Union Hospital, Tongji Medical College, Huazhong University of Science and Technology. These patients were randomly assigned to either a control group or an intervention group, with 30 patients in each group. Two groups implement routine care, and the intervention group implement integrated care by a “hospital-community-family” multidisciplinary team. After 12 weeks of intervention, the differences in frailty, cognition, depression scores, comprehension social support scores, and World Quality of Life Scale scores were compared between the two groups.

**Results:**

Before the intervention, there were no significant differences between the control group and the intervention group in terms of grip strength, step speed, frailty scores, cognitive function, depression scores, social support, and quality of survival. After the intervention, both groups showed improvements in grip strength and depression scores, with no significant differences observed between the groups. However, cognitive function, comprehension social support and quality of survival scores improved only in the intervention group. Notably, cognitive ability was the only outcome that showed a significant difference between the intervention group and the control group after 12 weeks of intervention (P < 0.05).

**Conclusions:**

Integrated care effectively enhances patients’ intrinsic capacity, with a notable improvement in cognitive abilities.

